# Oxidative stress enhances the immune response to oxidatively modified catalase enzyme in patients with Graves’ disease

**DOI:** 10.1002/jcla.23051

**Published:** 2019-10-16

**Authors:** Bochra Gargouri, Malek Mseddi, Fatma Mnif, Mohamed Abid, Hamadi Attia, Saloua Lassoued

**Affiliations:** ^1^ Faculty of Sciences of Sfax Tunisia Sfax Tunisia; ^2^ Laboratory LR11ES45 Research Group “Biotechnology and Pathology” National School of Engineers of Sfax Sfax Tunisia; ^3^ Department of endocrinology Hedi Chaker Hospital Sfax Tunisia

**Keywords:** autoantibody, catalase, Graves' disease, hydrogen peroxide, malondialdehyde, oxidative stress

## Abstract

**Background:**

Oxidative stress is associated with several autoimmune disorders and oxidative modification of proteins that may result in autoimmune response. This study aims to evaluate the catalase (CAT) activity and the autoimmune response against the native CAT and the oxidatively modified enzyme in patients with Graves' disease (GD) and healthy controls in a comparative way.

**Methods:**

The CAT activity was evaluated via spectrophotometric method. Using enzyme‐linked immunosorbent assay, the reactivities of autoantibody toward native, malondialdehyde (MDA) and hydrogen peroxide (H_2_O_2_) modified CAT were evaluated in plasmas of patients and controls.

**Results:**

Reduced CAT activity was found in patients compared with controls (*P* < .05). It was proved that levels of IgG antibodies against MDA‐modified CAT were higher than against unmodified ones (*P* < .001). No changes were found for the reactivities to H_2_O_2_‐modified CAT. Positive correlation was found between the reactivity to MDA‐modified CAT and the triiodothyronine level (*P* < .001, *r* = .6).

**Conclusion:**

Our findings incriminate the MDA in the autoantibodies reactivity to oxidatively modified CAT leading to a disturbed oxidative profile and/or the progression of GD pathology.

## 1 INTRODUCTION

Grave's disease (GD) is an autoimmune thyroid disorder that manifests infiltration of the thyroid gland with reactive lymphocytes. This lymphocytic infiltration leads to the activation of the thyrotropin receptor (TSH‐R)‐B‐lymphocytes that secrete anti‐thyrotropin receptor (TSH‐R) antibodies responsible for the stimulation of the thyroid gland causing the hyperthyroidism.^1^, ^2^ Consequently, further the autoimmune reaction, symptoms, and signs, such as weight loss, metabolic abnormalities, fatigue, heat intolerance, decreased appetite and cardiac manifestations, characterize persons with GD.^3^, ^4^, ^5^ Several studies on animals as well as on human tissues and plasmas provide evidences of an association between oxidative stress and GD.^6^, ^7^, ^8^, ^9^ During oxidative stress, reactive oxygen species (ROS) including hydrogen peroxide (H_2_O_2_), superoxide radical (O_2_^−•^) and hydroxyl radical (OH^•^) are generated and have inherent chemical properties that confer reactivity to different biological targets such as lipids, proteins, and deoxyribonucleic acid (DNA).^10^, ^11^ Usually, the body is able to defend itself against ROS by means of enzymes such as superoxide dismutase (SOD), catalase (CAT), and glutathione peroxidase (GPx). However, when the level of ROS exceeds the defense mechanisms, these enzymes may be itself oxidatively damaged.^12^ Indeed, anti‐CAT autoantibodies' production was described in human autoimmune pathologies such as systemic lupus erythematosus (SLE) and arthritis rheumatoid (AR).^13^ However, there is no report about the oxidative effects of ROS on CAT enzyme in thyroid disorders.

In this study, we determined whether oxidative modification of the CAT induced by MDA and H_2_O_2_ enhanced the reactivity of plasmas of patients with GD and healthy controls by comparison with the reactivity toward the native enzyme.

## 2 PATIENTS AND METHODS

### 2.1 Ethics approval

The performed experimental protocols and consent forms were in accordance with the guidelines of the Declaration of Helsinki and approved by the local Ethics Committee of the Hedi Chaker University Hospital of Sfax, Tunisia.

### 2.2 Patients recruitment

A total of 34 untreated Tunisian patients with GD were recruited from the department of endocrinology inpatient and outpatient clinics, Hedi Chaker University Hospital of Sfax, Tunisia.

In total, 65 healthy volunteers without familial history of autoimmune diseases and without any recent drug treatments were enrolled in the study (Tables 1 and 2).

**Table 1:** Basic clinical characteristics of Graves' disease patients and healthy controls

Parameters	Control	GD
Number	65	34
Age (y)	42.3 ± 9.8	42.3 ± 14.3
Gender (female/male)	51/14	31/3
FT4 (pg/mL)	11.2 ± 0.2	50.3 ± 5.8***
FT3 (pg/mL)	3.9 ± 0.01	14.8 ± 1.4***
TSH (µUI/mL)	1.5 ± 0.1	0.08 ± 0.01***
Anti‐TPO (UI/mL)	<50	1740.6 ± 78.2
Anti‐Tg (UI/mL)	<100	1710 ± 151.6
Anti‐R‐TSH (µUI/mL)	<2	37 ± 2.9

Note

Statistical analyses were performed using unpaired *t* test; there were no significant differences between the groups regarding the age (*P* > .05); and significant differences were found ***P < .001.

**Table 2:** Catalase activity in plasmas of Graves' disease patients

Test	Controls n = 65	GD n = 34
Catalase (U/mg of proteins)	8.73 ± 1.34	3.79 ± 0.76*

Note

The results are expressed as mean ± SEM Statistical analyses were performed using the unpaired *t* test, and significant differences were found *P < .05 by comparison with the control group.

### 2.3 Hormonal and antibodies tests

#### 2.3.1 Measurement of TSH

The evaluation of the TSH level was assessed by enzyme immunoassay using the ELFA technique (enzyme‐linked fluorescent assay). VIDAS TSH is an automated assay for the VIDAS system, which enables human TSH in serum or plasma to be quantitatively measured. The assay principle combines the indirect enzyme immunoassay method with a final fluorescent detection (ELFA). All reaction steps are performed by the VIDAS instrument. The disposable Solid Phase Receptacle serves both as a solid phase and as a pipetting device during the assay. Reagents for the assay are all contained in the sealed reagent strips. The results are expressed in µUI/mL.

#### 2.3.2 Measurement of FT3

The determination of the serum level of FT3 was carried out by a radioimmunoassay method using the RIA‐gnost®FT3 kit (CIS bio). This assay is based on a competition between a radioisotope‐labeled antigen and a cold antigen to be assayed for the same amount of antibody. At the bottom of each tube coated with Ac, 50 µL of sample and 1000 µL of the 125I‐FT3 tracer solution were successively deposited. After stirring for 120 minutes at room temperature and at a speed between 1120 and 3400 *g*, the liquid is removed by suction followed by taping on absorbent paper. The measurement of the radioactivity is carried out for one minute using a gamma counter (Wallac). For each series of samples, a calibration curve is established using standard solutions delivered with the kit. The results are expressed in pg/mL.

#### 2.3.3 Measurement of FT4

The RIA‐gnost® FT4 kit is used to evaluate free T4 using antibody‐coated tubes in a two‐step procedure. The serum sample is first incubated with a polyclonal antibody bound to the solid phase and then removed by decantation. Then, the radioactive tracer is added to lodge the sites of antibodies remaining free after decantation.

At the bottom of each tube coated with Ac, 100 μL of sample and 1 mL of incubation buffer are deposited successively. After stirring for 30 minutes using a horizontal stirrer at room temperature and at a speed between 1120 and 3400 *g*, the liquid is removed by suction followed by tapping on absorbent paper. Then, 1 mL of the 125I‐FT4 tracer solution is deposited and the tubes are stirred again for 60 minutes. After removing the remaining liquid that can adhere to the edge of the tubes by tapping, the measurement of radioactivity is carried out for one minute using a gamma counter (Wallac). For each series of samples, a calibration curve was established using standard solutions delivered with the kit. The results were expressed in pg/mL.

#### 2.3.4 Measurement of anti‐Tg and anti‐TPO

The search for anti‐thyroid antibodies (anti‐Tg and anti‐TPO) was performed by an ELISA kit (Orgentec). The microwells are sensitized with recombinant TPO or human TG. In a first step, 100 μL of each calibrator of the positive and negative controls and sera diluted 1: 100th in the dilution buffer (supplied with the kit) are deposited in the appropriate microwells, thus allowing the specific binding of the antibody to the fixed antigen. After incubation for 30 minutes at room temperature, the plate is washed three times with a volume of washing buffer (supplied with the kit) of between 250 and 300 μL per well manually. Subsequently, 100 µL of conjugate (anti‐human IgG purified and labeled with peroxidase and ready for use) is deposited in each microwell and incubated at room temperature for 30 minutes. During incubation, these labeled antibodies bind to IgG that has recognized TG or TPO. Excess labeled unbound conjugate is removed by three further washes. Subsequently, 100 µL of the solution of TMB (3,3,5,5 'tetramethylbenzidine) is added. A blue color is obtained and whose intensity is proportional to the concentration of autoantibodies in the sample. The reaction is stopped by the addition of a hydrochloric acid solution (1 mol/L). The optical density of each well is read at 450 nm using a bichromatic plate reader (Labsystems Multiskan) within 30 minutes after stopping the reaction.

### 2.4 Catalase activity

The method of Aebi 1984 was used to evaluate the CAT activity.^14^

### 2.5 Oxidative modification of the catalase enzyme

The CAT modification was assessed according to the method of MSEDDI at al. 2017.^7^ Two modifications of the CAT enzyme were assessed by the MDA and the H_2_O_2_. After acid hydrolysis of the 1,1,3,3‐tetraethoxypropane, the obtained MDA was brought to pH 7 using KOH (1 N). This MDA was incubated with CAT enzyme (1.8 mg/mL in filtered PBS) for 24 hours at 37°C. A micro bio‐spin column (Bio‐Rad) 30 kd was used to eliminate the unbound MDA via a centrifugation (16 000 *g*, 20 minutes). Concerning the oxidation by the H_2_O_2_, 1.8 mg/mL of the CAT was incubated for one hour with UV in the presence of H_2_O_2_ (100 mmol/L). Enzyme modifications were verified by the dosage of the MDA level (0 vs 0.25 µmol/mL) and the carbonyl group (1.9 vs 5.86 nmol/mL).

### 2.6 Enzyme‐linked immunosorbent assay (ELISA)

The CAT modification was assessed according to the method of MSEDDI et al. 2017.^7^ The optical density (OD) at 405 nm was determined using a micro‐ELISA plate reader.

### 2.7 Protein quantification

Protein Assay Kit from Bio‐Rad (France) was used to determine the protein level in the plasmas of patients and controls. The bovine serum albumin served as a standard.

The plasmas are diluted in a volume of 800 µL of sterile water and then added with 200 µL of Bradford reagent. The optical density (OD) is read at 595 nm.

### 2.8 Statistical analyses

The evaluation of differences between patients and controls regarding the plasmatic CAT activity and the MDA level was performed using the unpaired *t* test. The differences in the plasmatic reactivity to the native CAT between patients and healthy controls were assessed by ANOVA test. The evaluation of differences in plasmatic reactivities toward native and modified CAT was considered by the paired *t* test. The correlation study was assessed using Pearson correlation test. All data are presented as means ± standard error mean (SEM). Statistical analysis and figures were performed using GraphPad Prism 6 program.

## 3 RESULTS

### 3.1 Evaluation of hormonal and antibodies levels

The evaluation of the level of the thyroid hormones in GD patients' showed an increase in FT4 and FT3 concomitant with reduced TSH in comparison with healthy controls (*P* < .001) (Table 1). In addition, high rates of anti‐thyroid autoantibodies, anti‐TPO, anti‐Tg, and anti‐TSH‐R, were detected in patients compared with controls (Table 1).

### 3.2 Determination of the catalase activity

The plasmatic CAT activity was significantly reduced in patients compared with controls (*P* < .05) (Table 2).

### 3.3 Immune response to MDA and H_2_O_2_ modified catalase

**Figure 1:**
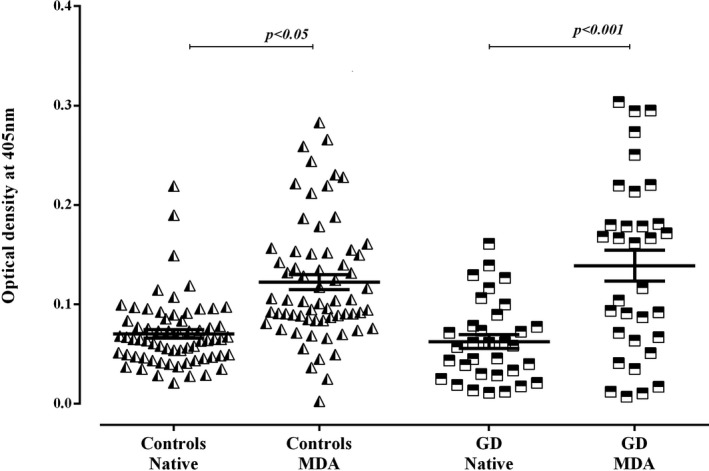
Reactivities of plasmas to malondialdehyde‐modified catalase in patients with Graves' disease and controls. Data are presented as mean values of optical density at 405 nm (±SEM) of experiments performed in duplicate. Statistical analyses were performed using paired *t* test, and significant differences were found ***P < .001

**Figure 2:**
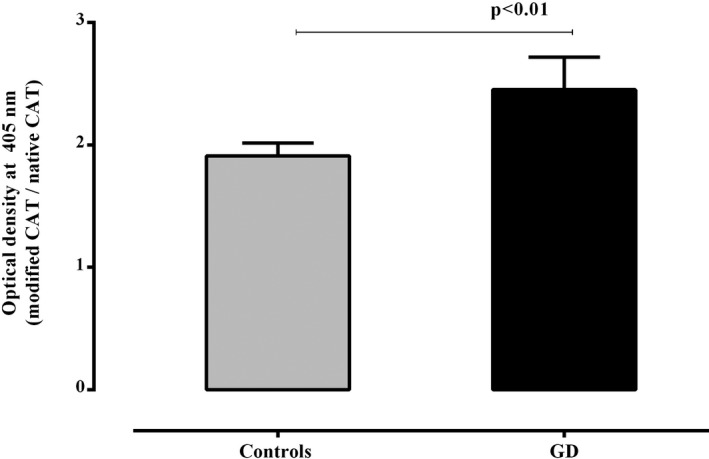
Comparison of the ratio of reactivity to malondialdehyde‐modified catalase/reactivity to native catalase in patients with Graves' disease (GD) and controls. Data are presented as ratio of mean values of optical density at 405 nm (±SEM) of reactivities toward modified catalase and those toward the native one. Statistical analyses were performed using unpaired *t* test

**Figure 3:**
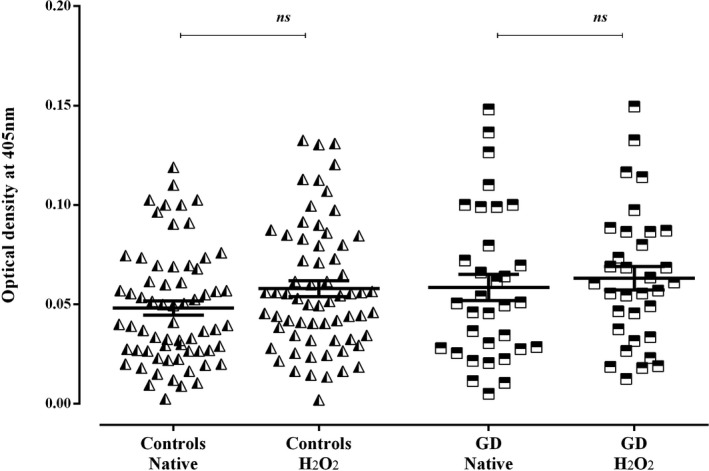
Reactivities of plasmas to hydrogen peroxide–modified catalase in patients with Graves' disease and controls. Data are presented as mean values of optical density at 405 nm (±SEM) of experiments performed in duplicate. Statistical analyses were performed using paired *t* test, there were no significant differences *P* > .05

The plasmatic reactivity toward MDA‐modified CAT was higher than the native one in patients with GD (*P* < .001) (Figure 1). Remarkably, a slight increase in the reactivity toward MDA‐modified CAT was also noted in controls (*P* < .05) (Figure 1). Nevertheless, the ratio (OD of MDA‐modified‐CAT/ OD of native CAT) of the control group remains significantly inferior to that observed in patients (*P* < .05) (Figure 2). Concerning the modification of the CAT using the H_2_O_2_, no changes were noted in the reactivity to the H_2_O_2_‐modified CAT in comparison with the native form in patients with GD as well as in controls (Figure 3).

### 3.4 Correlation study

**Figure 4:**
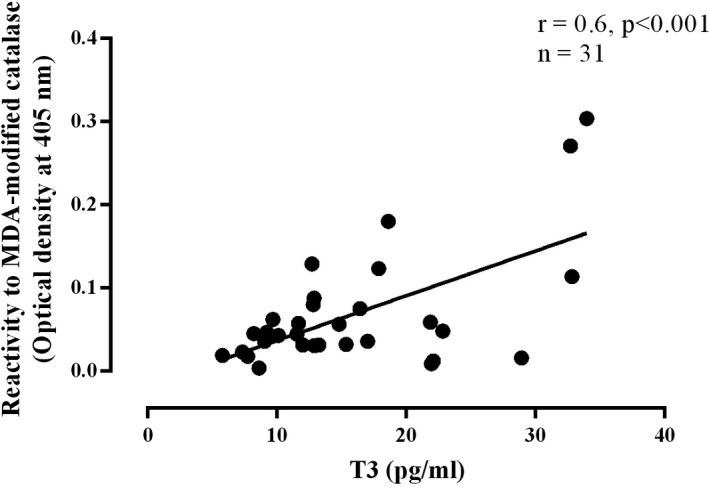
Correlation between the FT3 level and the reactivity to the malondialdehyde‐modified catalase

The statistical analyses showed a significantly positive correlation between the reactivity toward the MDA‐modified CAT and the level of FT3 hormone (*r* = .6, *P* < .001, n = 31) (Figure 4).

## 4 DISCUSSION

Results reported in the present study demonstrate that the CAT activity was reduced in plasmas of patients with GD compared to controls. These data are consistent with several studies also showing a decrease in the CAT activity in sera and plasma of patients with GD.^16^, ^17^ So, the reduced activity of the enzyme may indicate an excessive consumption or structural alterations due to oxidative stress. In fact, oxidative modifications may alter the function of the enzyme and/or create new epitopes that could generate antibody response. Several studies supporting this hypothesis showed that the CAT enzyme is a major protein target of the lipid peroxidation product 4‐HNE in autoimmune diseases such as the SLE.^18^ In order to verify our hypothesis, we evaluate in a comparative way the reactivity of plasmas of patients with GD and healthy controls toward the native CAT and toward the MDA‐ and H_2_O_2_‐modified enzyme. Our findings revealed high levels of IgG anti‐CAT antibodies in patients compared with controls. This result could be explained by the autoimmune background in GD. The evaluation of the reactivity of patients' plasmas against the H_2_O_2_‐modified CAT showed no changes compared with the reactivity toward the native CAT. This result indicates that the CAT enzyme is endowed with resistance to the H_2_O_2_ since it represents its substrate. Indeed, the CAT enzyme is usually endowed with a molecule of NADH, H +, which protects it from a possible inactivation by the H_2_O_2_.^19^, ^20^ Just like the H_2_O_2_, the CAT enzyme was oxidatively modified by MDA and the immune response was evaluated in patients and controls. Our results revealed enhanced reactivity to the MDA‐modified CAT as compared to the reactivity toward the native one in patients. Also, this high reactivity to the MDA‐modified CAT correlates positively with the level of the thyroid hormone triiodothyronine. Taken together, these observations suggest that the high production of the H_2_O_2_ during the hormonogenesis process in GD is not directly involved in the alteration of the function of the CAT, but rather indirectly through the generation of lipid peroxidation products such as the MDA that severely affects its activity. In fact, previous reports have implicated thyroid hormone hypersecretion in the oxidative stress establishment. The hyperthyroidism state was known to be the origin of the increase of the basal metabolic state and the mitochondrial respiration process, resulting in increased generation of superoxide (O_2_^•−^). This radical can lead to the formation of many other reactive species, including the hydroxyl radicals (OH^•^), which can readily start the free radical process of lipid peroxidation.^6^ Also, thyroid H_2_O_2_ involved in the hormone biosynthesis can be turned into the highly reactive hydroxyl radical (OH^•^) via the Fenton reaction.^21^, ^22^, ^23^ The highly reactive OH^•^ is responsible for the abstraction of the hydrogen from lipids that results in the formation of lipid alkyl radical during the initiation of the lipid peroxidation process. During the termination step, unsaturated reactive aldehydes, like MDA and other products, were generated.^24^, ^25^ Indeed, the MDA, one of the most abundant lipid peroxidation–derived aldehydes, can easily diffuse across membranes and can covalently modify proteins in the cytoplasm and the nucleus, far from their site of origin.^26^ Moreover, it can bind covalently with proteins to form MDA‐modified protein adducts that are immunogenic and may act as neo‐autoantigens in initiating a strong autoimmune response.^27^, ^28^, ^29^ In line with our results, studies by Ben Mansour et al (2008, 2010) demonstrate that the MDA‐modified CAT and SOD were better recognized in sera of patients with two autoimmune pathologies than the unmodified form.^13^, ^15^ Besides, Bakala et al (2012) demonstrated that under an oxidative stress condition, the CAT enzyme is more susceptible than other antioxidant enzyme to be affected by the glycation stress in mitochondrial liver extract of rat.^30^

The heightened reactivity of IgG autoantibodies against MDA‐modified CAT was evidenced in plasmas of patients with GD. It seems that the MDA has a key role in the appearance of new epitopes that could be the target of immune response, which could contribute to the misbalance of the oxidative profile and/or the development of complications of the pathology.

## References

[1] Parish NM , Cooke A . Mechanisms of autoimmune thyroid disease. Annu Rev Pathol. 2004;1(3):147‐156. 10.1146/annurev-pathol-012513-104713 PMC4128637 24460189

[2] Eschler DC , Hasham A , Tomer Y . Cutting edge: the etiology of autoimmune thyroid diseases. Clin Rev Allergy Immunol. 2011;41:190‐197. 21234711 10.1007/s12016-010-8245-8 PMC3129418

[3] Şahlı E , Gündüz K . Thyroid‐associated Ophthalmopathy. Türk Oftalmoloji Dergisi. 2017;47:94‐105. 10.4274/tjo.80688 PMC5384127 28405484

[4] Terry J , Smith MD , Laszlo Hegedüs MD . Graves' disease. N Engl J Med. 2016;375:1552‐1565. 27797318 10.1056/NEJMra1510030

[5] Melpomeni P , Grigoria B , Dimitriadis G . Lipid abnormalities and cardiometabolic risk in patients with overt and subclinical thyroid disease. J Lipids. 2011;2011:575840. 21789282 10.1155/2011/575840 PMC3140027

[6] Lassoued S , Mseddi M , Mnif F , et al. A comparative study of the oxidative profile in Graves' disease, Hashimoto's thyroiditis, and papillary thyroid cancer. Biol Trace Elem Res. 2010;138:107‐115. 20204550 10.1007/s12011-010-8625-1

[7] Mseddi M , Ben Mansour R , Gargouri B , et al. Proteins oxidation and autoantibodies' reactivity against hydrogen peroxide and malondialdehyde ‐oxidized thyroid antigens in patients' plasmas with Graves' disease and Hashimoto Thyroiditis. Chem Biol Interact. 2017;272:145‐152. 28431875 10.1016/j.cbi.2017.04.013

[8] Zarkovic M . The role of oxidative stress on the pathogenesis of Graves' disease. J Thyroid Res. 2012;2012:302537. 22175033 10.1155/2012/302537 PMC3235898

[9] Mseddi M , Ben Mansour R , Gouiia N , et al. A comparative study of nuclear 8‐hydroxyguanosine expression in Autoimmune Thyroid Diseases and Papillary Thyroid Carcinoma and its relationship with p53, Bcl‐2 and Ki‐67 cancer related proteins. Adv Med Sci. 2017;62:45‐51. 28187375 10.1016/j.advms.2016.06.003

[10] Schieber M , Chandel NS . ROS Function in redox signaling and oxidative stress. Curr Biol. 2014;24:R453‐R462. 24845678 10.1016/j.cub.2014.03.034 PMC4055301

[11] Jones DP . Radical‐free biology of oxidative stress. Am J Physiol Cell Physiol. 2008;295:C849‐C868. 18684987 10.1152/ajpcell.00283.2008 PMC2575825

[12] Gerling IC . Oxidative stress, altered‐self and autoimmunity. Open Autoimmunity J. 2009;1:33‐36.

[13] Ben Mansour R , Lassoued S , Gargouri B , El Gaied A , Attia H , Fakhfakh F . Increased levels of autoantibodies against catalase and superoxide dismutase associated with oxidative stress in patients with rheumatoid arthritis and systemic lupus erythematosus. Scand J Rheumatol. 2008;37:103‐108. 18415766 10.1080/03009740701772465

[14] Aebi H . Catalase in vitro. Methods Enzysmol. 1984;105:121‐126. 10.1016/s0076-6879(84)05016-3 6727660

[15] Ben Mansour R , Lassoued S , Elgaied A , et al. Enhanced reactivity to malondialdehyde‐modified proteins by systemic lupus erythematosus autoantibodies. Scand J Rheumatol. 2010;39:247‐253. 20429675 10.3109/03009740903362511

[16] Marcocci C , Leo Maria M , Altea A . Oxidative Stress in Graves' disease. Eur Thyroid J. 2012;1:80‐87. 24783001 10.1159/000337976 PMC3821469

[17] Wassmann S , Wassmann K , Nickenig G . Modulation of oxidant and antioxidant enzyme expression and function in vascular cells. Hypertension. 2004;44:381‐386. 15337734 10.1161/01.HYP.0000142232.29764.a7

[18] D'souza A , Kurien BT , Rodgers R , et al. Detection of catalase as a major protein target of the lipid peroxidation product 4‐HNE and the lack of its genetic association as a risk factor in SLE. BMC Med Genet. 2008;7:62. 10.1186/1471-2350-9-62 PMC2474584 18606005

[19] Kodydkova J , Vavrova L , Kocik M , Zak A . Human catalase, its polymorphisms, regulation and changes of its activity in different diseases. Folia Biol (Praha). 2014;60:153‐167. 25152049 10.14712/fb2014060040153

[20] Putnam CD , Arvai AS , Bourne Y , Tainer JA . Active and inhibited human catalase structures: ligand and NADPH binding and catalytic mechanism. J Mol Biol. 2000;296:295‐309. 10656833 10.1006/jmbi.1999.3458

[21] Carvalho DP , Dupuy C . Role of the NADPH Oxidases DUOX and NOX4 in thyroid oxidative stress. Eur Thyroid J. 2013;2:160‐167. 24847449 10.1159/000354745 PMC4017742

[22] Rinnerthaler M , Bischof J , Streubel MK , Trost A , Richter K . Oxidative stress in aging human skin. Biomolecules. 2015;5:545‐589. 25906193 10.3390/biom5020545 PMC4496685

[23] Venditti P , De Rosa R , Di Meo S . Effect of thyroid state on H2O2 production by rat liver mitochondria. Mol Cell Endocrinol. 2003;31:185‐192. 10.1016/s0303-7207(02)00332-5 12890580

[24] Shah D , Mahajan N , Sah S , Nath SK , Paudyal B . Oxidative stress and its biomarkers in systemic lupus erythematosus. J Biomed Sci. 2014;17(21):23. 10.1186/1423-0127-21-23 PMC3995422 24636579

[25] Rác M , Křupka M , Binder S , et al. Oxidative damage of U937 human leukemic cells caused by hydroxyl radical results in singlet oxygen formation. PLoS ONE. 2015;10(3):e0116958. 25730422 10.1371/journal.pone.0116958 PMC4346403

[26] Ayala A , Muñoz MF , Argüelles S . Lipid peroxidation: production, metabolism, and signaling mechanisms of malondialdehyde and 4‐hydroxy‐2‐nonenal. Oxid Med Cell Longev. 2014;2014:360438. 24999379 10.1155/2014/360438 PMC4066722

[27] Wang G , Wang J , Fan X , Ansari GA , Khan MF . Protein adducts of malondialdehyde and 4‐hydroxynonenal contribute to trichloroethene‐mediated autoimmunity via activating Th17 cells: dose‐ and time‐response studies in female MRL+/+ mice. Toxicology. 2012;292:113‐122. 22178267 10.1016/j.tox.2011.12.001 PMC3264691

[28] Pizzimenti S , Ciamporcero E , Daga M , et al. Interaction of aldehydes derived from lipid peroxidation and membrane proteins. Front Physiol. 2013;4:242. 24027536 10.3389/fphys.2013.00242 PMC3761222

[29] Negre‐Salvayre A , Coatrieux C , Ingueneau C , Salvayre R . Advanced lipid peroxidation end products in oxidative damage to proteins. Potential role in diseases and therapeutic prospects for the inhibitors. Br J Pharmacol. 2008;153:6‐20. 17643134 10.1038/sj.bjp.0707395 PMC2199390

[30] Bakala H , Hamelin M , Mary J , Borot‐Laloi C , Friguet B . Catalase, a target of glycation damage in rat liver mitochondria with aging. Biochim Biophys Acta. 2012;1822:1527‐1534. 22683338 10.1016/j.bbadis.2012.05.016

